# Topical Fibronectin Improves Wound Healing of Irradiated Skin

**DOI:** 10.1038/s41598-017-03614-y

**Published:** 2017-06-20

**Authors:** Maxwell B. Johnson, Brandon Pang, Daniel J. Gardner, Solmaz Niknam-Benia, Vinaya Soundarajan, Athanasios Bramos, David P. Perrault, Kian Banks, Gene K. Lee, Regina Y. Baker, Gene H. Kim, Sunju Lee, Yang Chai, Mei Chen, Wei Li, Lawrence Kwong, Young-Kwon Hong, Alex K. Wong

**Affiliations:** 1 Division of Plastic and Reconstructive Surgery, Keck School of Medicine of USC, 1510 San Pablo St., Suite 415, Los Angeles, CA 90033 USA; 2 Department of Surgery, Keck School of Medicine of USC, 1510 San Pablo St., Suite 415, Los Angeles, CA 90033 USA; 3 Department of Dermatology, Keck School of Medicine of USC, 1510 San Pablo St., Suite 415, Los Angeles, CA 90033 USA; 4 Department of Biochemistry and Molecular Biology, Keck School of Medicine of USC, 1510 San Pablo St., Suite 415, Los Angeles, CA 90033 USA; 5 Norris Comprehensive Cancer Center, Keck School of Medicine of USC, 1510 San Pablo St., Suite 415, Los Angeles, CA 90033 USA; 6 Center for Craniofacial Molecular Biology, Ostrow School of Dentistry, University of Southern California, 2250 Alcazar St., Los Angeles, 90089 CA USA; 7 Department of Translational Pathology, MD Anderson Cancer Center, 1515 Holcombe Blvd, Houston, TX 77030 USA

## Abstract

Wound healing is significantly delayed in irradiated skin. To better understand global changes in protein expression after radiation, we utilized a reverse phase protein array (RPPA) to identify significant changes in paired samples of normal and irradiated human skin. Of the 210 proteins studied, fibronectin was the most significantly and consistently downregulated in radiation-damaged skin. Using a murine model, we confirmed that radiation leads to decreased fibronectin expression in the skin as well as delayed wound healing. Topically applied fibronectin was found to significantly improve wound healing in irradiated skin and was associated with decreased inflammatory infiltrate and increased angiogenesis. Fibronectin treatment may be a useful adjunctive modality in the treatment of non-healing radiation wounds.

## Introduction

Although radiation therapy is an important part of the treatment of solid tumors, it has dose-limiting ill effects on normal tissues. Skin is particularly prone to radiation injury because resident cells are rapidly dividing^1^. Chronic radiation skin injury is characterized by dermal atrophy, fibrosis, vascular damage, chronic ulceration, and poor wound healing^2^. While the deleterious effects of ionizing radiation on wound healing are well-described, further mechanistic studies have the potential to expand our armamentarium of treatment modalities for non-healing radiation wounds^3, 4^.

The principles of wound healing follow an orderly sequence of three phases: inflammation, proliferation, and remodeling. However, radiation impairs this sequence, inhibiting the normal wound healing process. During the inflammatory phase, tissue levels of various cytokines and chemokines involved in normal wound healing, including VEGF, TGF-beta, TNF-alpha and IFN-gamma, are deranged^5^. Additionally, the generation of reactive oxygen species leads to endothelial damage and dysfunction, producing progressive vasculopathy and impairing the formation of granulation tissue, re-epithelialization, and neovascularization that characterizes the proliferative phase^6^. Moreover, fibroblasts, which comprise a crucial role in the remodeling phase of collagen deposition and remodeling, produce highly disorganized collagen framework leading to impaired wound strength^7, 8^. The culmination of these negative effects of radiation on wound healing manifests clinically as atrophic, dry, dyspigmented skin that is commonly fibrotic and/or ulcerated, healing poorly or not at all^9^.

The mechanism of radiation-induced skin fibrosis is a complex, and involves terminal differentiation of fibroblasts, abnormal collagen deposition with loss of adnexal structures, disordered vasculature, and dysfunctional chronic inflammation. The constitution and function of dermal extracellular matrix (ECM) is critical to wound healing^10^. Irradiation results in permanent and intrinsic damage to fibroblasts, the primary cell type responsible for the production of ECM^11, 12^. These changes are associated with characteristic alterations in ECM protein composition and breaking strength, both acutely and over time^8, 13^.

In the present study, we performed a proteomic analysis of paired irradiated and normal samples of skin obtained from human patients undergoing post-oncologic reconstruction. We found that fibronectin was among the most significantly downregulated proteins in irradiated skin. Fibronectin is an ECM glycoprotein that is involved in a number of cellular mechanisms important to wound healing, including cell growth and migration, and serves as a binding site for a number of growth factors^14–16^. It promotes wound healing *in vivo*, and is protective against irradiation *in vitro* ^17–21^. Additionally, there is some evidence to suggest that fibronectin levels are altered after exposure to ionizing radiation in non-skin tissues^22–24^. To date there has been no evaluation of fibronectin levels in human skin that has been irradiated *in vivo*.

Using a murine wound model, we demonstrated that fibronectin is downregulated in response to radiation in mouse skin. Furthermore, topical fibronectin gel led to significantly improved healing in irradiated wounds. This was associated with a significant reduction in acute inflammation and an increase in angiogenesis. These findings suggest that fibronectin may be involved in the pathogenesis of poor wound healing after radiation skin injury, and that exogenous supplementation may assist in the repair of radiation-damaged tissue.

## Results

**Figure 1:**
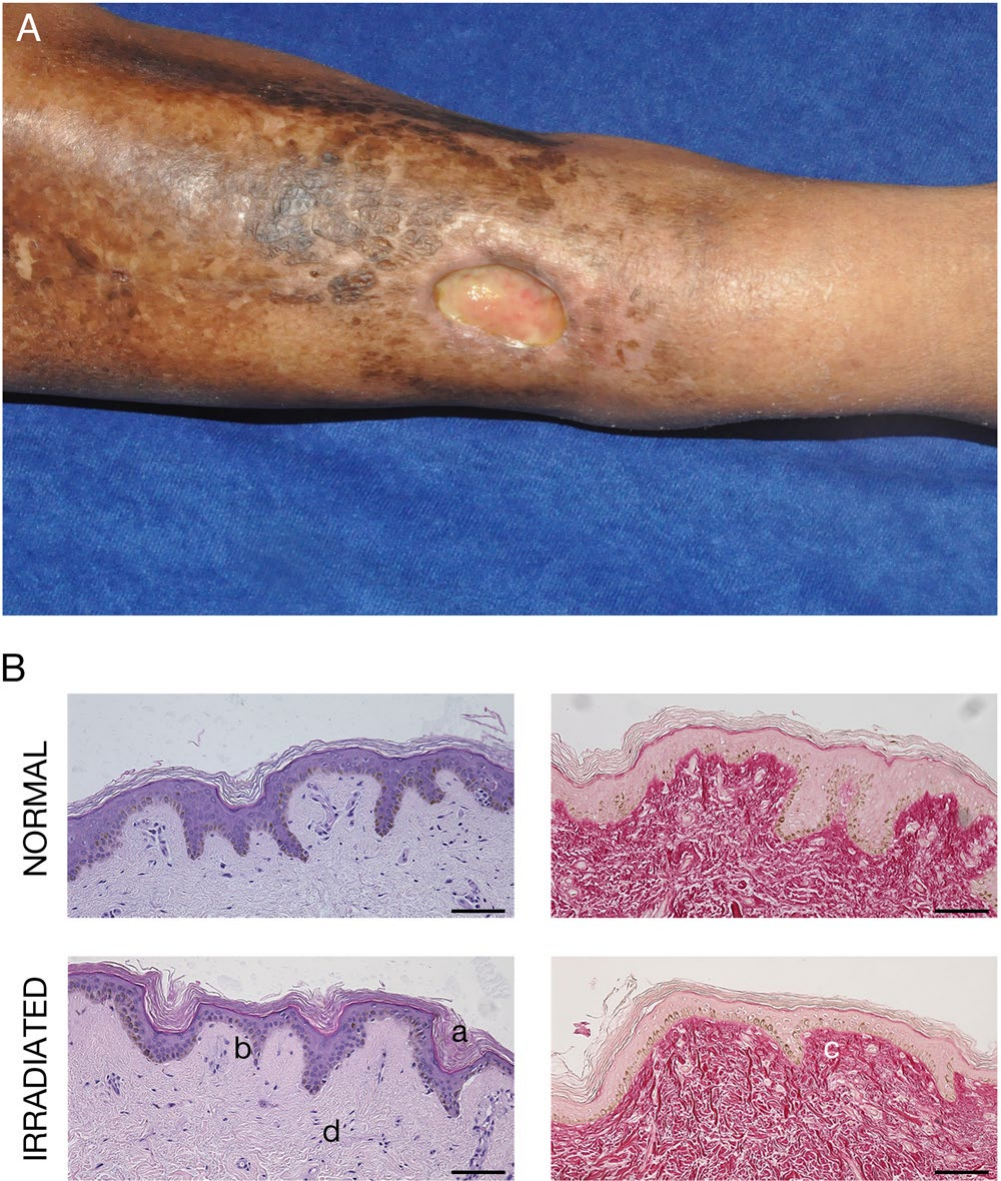
(**A**) Photo of a patient with a non-healing lower extremity wound. Note the skin discoloration associated with radiation injury. (**B**) Representative example of H&E (left) and Sirius red (right) staining of paired normal (top) and irradiated (bottom) human skin samples, demonstrating abnormally thick stratum corneum (a), thin epidermis (b), disordered collagen (c), and chronic inflammatory cells (d) characteristic of radiation-induced skin damage. Scale bars represent 100 µm.

Paired irradiated and non-irradiated skin samples were obtained under an IRB-approved protocol from patients who had undergone radiation for the treatment of either breast cancer or sarcoma (Fig. 1A). In all cases, a total dose of 40–60 Gray was administered at least 6 months prior to harvest. Histological analysis of these paired samples demonstrated classic findings associated with chronic radiodermatitis, including hyperkeratosis, epidermal atrophy, fibrosis, and chronic inflammatory infiltrate (Fig. 1B).

**Figure 2:**
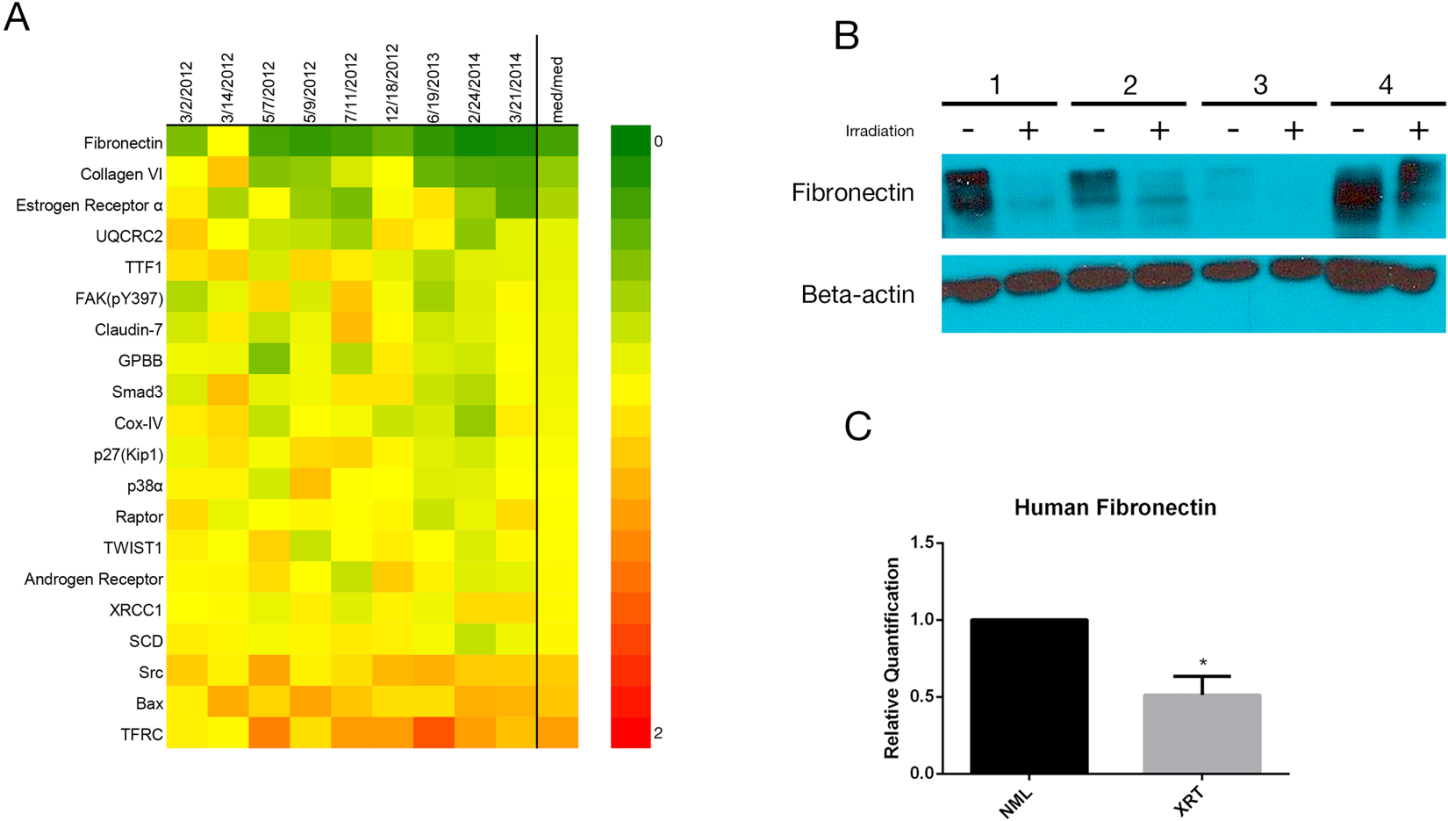
(**A**) Heat map of reverse phase protein array of paired irradiated and non-irradiated human skin samples. Samples are listed by date collected and labeled NML (normal) or XRT (radiated). Green corresponds to higher and red to lower relative expression. (**B**) Cropped Western blot of fibronectin expression in four pairs of human skin samples. (**C**) Relative quantification of fibronectin gene expression in paired human skin samples by qRT-PCR.

To assess whether there is a pattern of protein dysregulation in radiation-damaged skin, we utilized a proteomic approach known as Reverse Phase Protein Array (RPPA)^25^. The expression of 210 proteins was quantitatively analyzed. Of those proteins, 20 were significantly differentially expressed (*p* < 0.05). A heat map of these proteins is depicted in Fig. 2A. A number of these proteins—fibronectin, collagen VI, p27(Kip1), and androgen receptor—are relevant to skin function. Fibronectin exhibited the greatest fold-change of 0.3. We confirmed this result with Western blotting and quantitative RT-PCR of four additional pairs of samples (Fig. 2B,C, Supplementary Figure 1).

**Figure 3:**
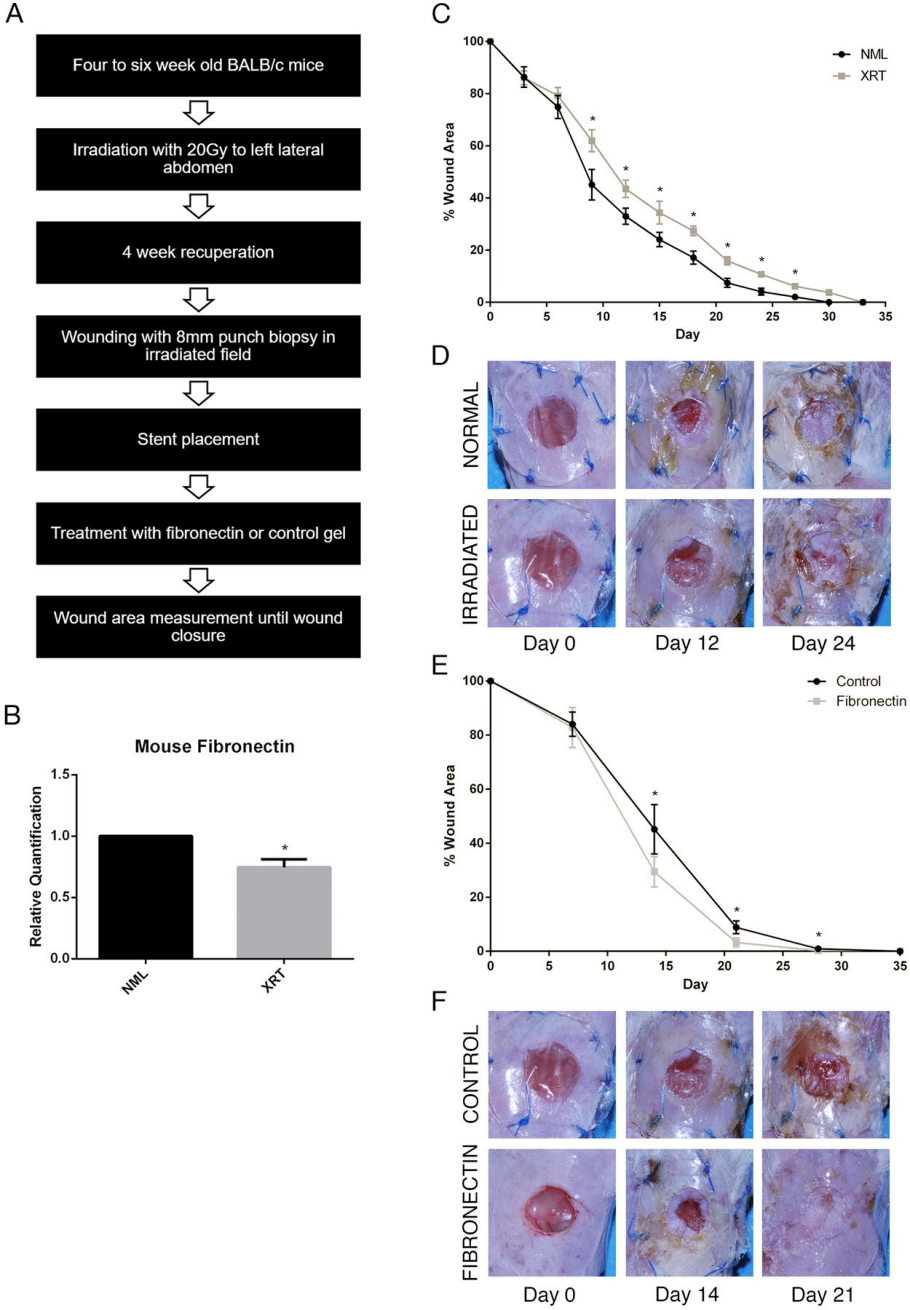
(**A**) Flow diagram for experimental methodology. (**B**) qRT-PCR demonstrating that fibronectin expression is significantly downregulated after radiation of mouse skin 4–6 weeks after exposure. (**C**) Irradiated skin heals significantly slower than normal skin at all time points (p < 0.05). (**D**) Representative normal (top) and irradiated (bottom) wounds at days 0, 12, and 24 post-wounding. (**E**) Topical application of fibronectin at the time of wounding is associated with significantly accelerated wound healing at all time points (p < 0.05). (**F**) Representative radiated wounds with vehicle control (top) and fibronectin treatment (bottom) at days 0, 14, and 21.

To further investigate the relationship between radiation and fibronectin expression in skin, we utilized a murine model of chronic radiation skin injury. Balb/c mice were exposed to 20 Gy irradiation on one dorsal side and allowed to recover for 4 weeks (Fig. 3A). Skin was then biopsied from the non-irradiated and irradiated sides for analysis. Histology revealed typical phenotypic changes after radiation including dermal atrophy and chronic inflammation (data not shown). Quantitative RT-PCR demonstrated that fibronectin was significantly downregulated in mouse irradiated skin (*p* < 0.05) (Fig. 3B). Next, to confirm that irradiation was associated with delayed wound healing, paired 8mm irradiated and non-irradiated full-thickness skin wounds were created and followed for over 30 days (n = 14). Throughout this entire period, the irradiated wounds exhibited delayed healing, with the maximum difference at day 9 (Fig. 3C,D).

**Figure 4:**
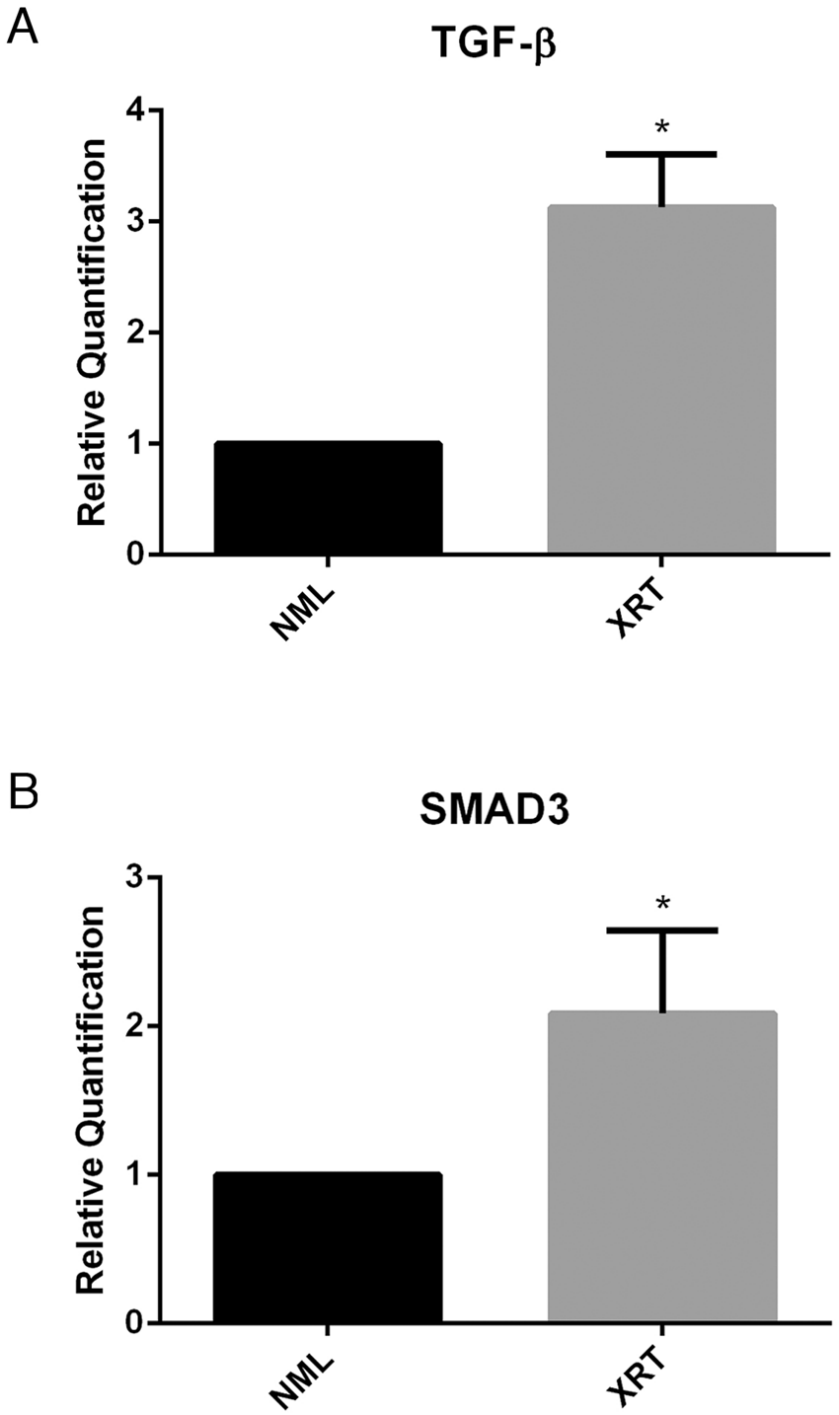
Relative quantification of (**A**) TGF-β and (**B**) SMAD3 expression in irradiated and non-irradiated murine skin samples, quantified by RT-PCR.

In order to assess the impact of fibronectin supplementation on irradiated wound healing, we next created unilateral irradiated wounds. Mice receiving a total dose of 2.0 mg of topical fibronectin directly on their wounds healed significantly faster than controls (Fig. 3E,F). Mean percent wound area remaining at day 14 was 45.2% in the control group, as compared to 29.4% in the fibronectin group (*p* < 0.05). Similarly, at day 21, mean percent wound area remaining was 8.9% for control animals, compared to 3.2% for mice treated with topical fibronectin (*p* < 0.05). To further validate the irradiated wound healing model, wounds were biopsied at day 18 and analyzed for genes that are known to be associated with radiation-induced fibrosis and poor wound healing^26, 27^. Transforming growth factor beta (TGF-β) and its related signal transducer SMAD3 were upregulated 3-fold and 2-fold, respectively, compared to non-irradiated wounds (Fig. 4A,B).

**Figure 5:**
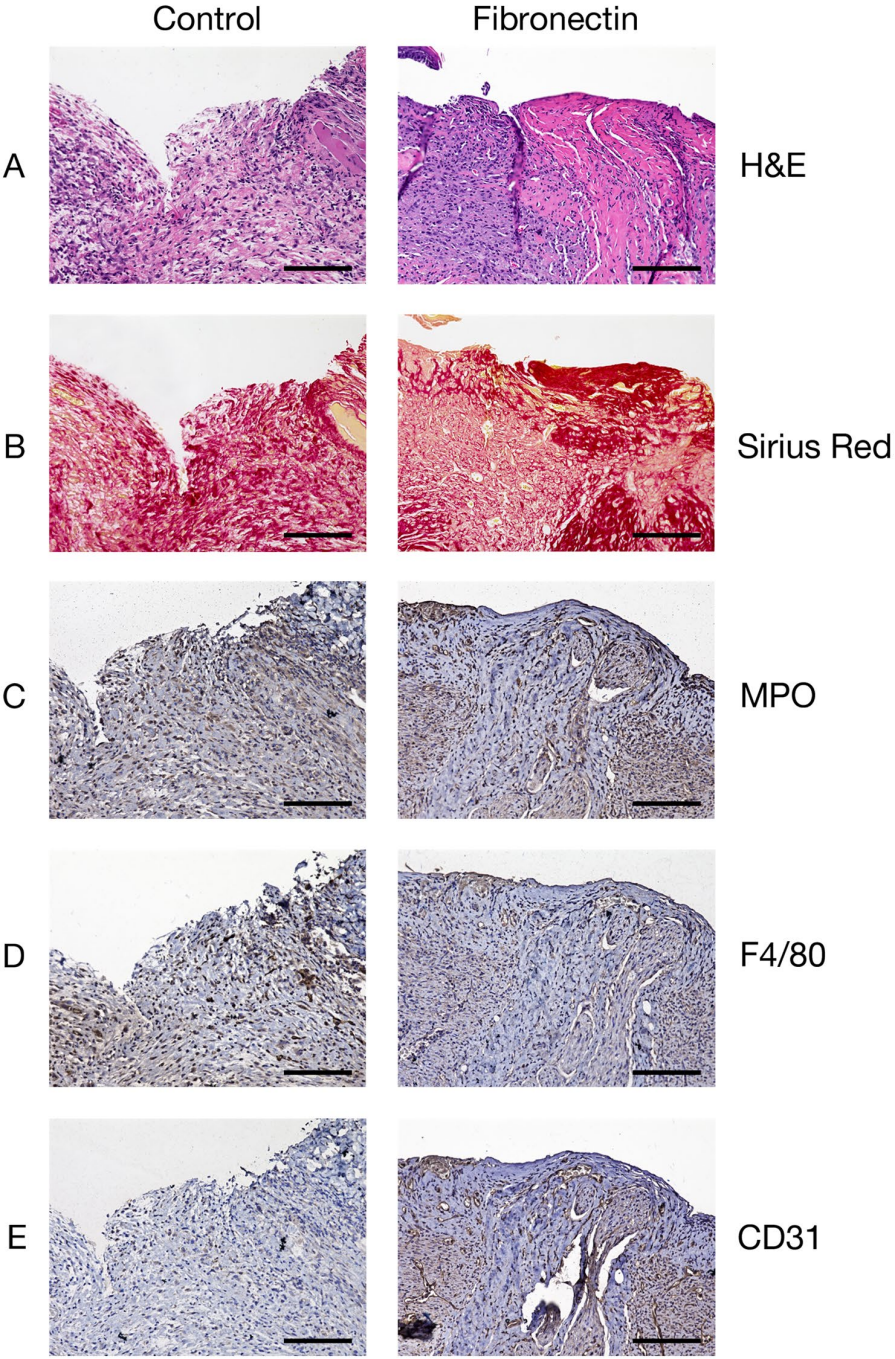
Histological analysis of control and fibronectin-treated radiated wounds. (**A**) H&E. (**B**) Sirius red reveals increased fascicular and horizontal collagen deposition in fibronectin-treated wounds. (**C**) Anti-MPO IHC shows fewer neutrophils in fibronectin-treated wounds. (**D**) Anti-F4/80 IHC demonstrates fewer macrophages in fibronectin-treated wounds. (**E**) Anti-CD31 IHC demonstrates increased endothelial cell presences in fibronectin-treated wounds. Scale bars represent 50 µm.

Wounds were harvested at day 15 to characterize the mid-healing microenvironment after treatment with fibronectin. Hematoxylin and eosin and Sirius Red stains demonstrated that wounds receiving fibronectin were more structurally organized with less inflammatory infiltrate, compared to control wounds that appeared more fibrotic and hypercellular (Fig. 5A,B). Anti-myeloperoxidase (MPO) and anti-F4/80 immunohistochemistry revealed reduced infiltration of neutrophils and macrophages (Fig. 5C,D). Fibronectin-treated wounds also had increased angiogenesis, confirmed by anti-CD31 immunohistochemistry (Fig. 5E). Fibronectin-treated wounds had a healing score of 15 (fair healing), while control wounds had a score of 9 (poor healing)^28^.

## Discussion

Complications of radiotherapy pose a significant problem for patients and physicians. Since the mechanism of pathogenesis for radiation skin injury has not been fully elucidated, few targeted therapies exist for this problem in clinical practice. Microvascular injury and subsequent tissue hypoxia is widely touted as the primary mechanism by which ionizing radiation causes chronic skin injury^29^. The hypoxic environment can facilitate elevated expression of TGF-β, a cytokine implicated in radiation-induced fibrosis and poor wound healing^30^. Although there is some evidence to suggest progressive intimal proliferation leading to permanent obliterative endarteritis, key studies have demonstrated normal oxygen tension in both animal and human skin with late-stage severe radiation injury^31–33^. This suggests that microvascular damage and tissue hypoxia may be transient, fueling research on the impact of radiation on other skin-specific factors.

The dermal extracellular matrix and its constituent cells and proteins are critical to skin homeostasis, skin pathology, and wound healing. ECM functions are mediated by a wide variety of mechanisms, including providing a suitable microenvironment for resident cells and binding and/or releasing important growth factors^10^. As a result, irradiation’s effect on fibroblasts and associated ECM production are frequent areas of research. Ionizing radiation has been shown to result in diminished growth and function of fibroblasts independent of blood supply^11, 12^. These alterations coincide with an acute reduction in collagen and wound breaking strength, followed by late-phase overexpression of collagen^8, 13^. Implantation of non-irradiated dermal fibroblasts can reverse many of these changes, improving wound tensile strength, toughness, and other mechanical measures^34^. In short, ionizing radiation has a profound impact on the dermal ECM, correlating directly to wound healing and strength.

In this report, we utilized a proteomic approach to identify radiation-induced skin changes (Fig. 2A). Out of 210 proteins in our screen, 20 achieved significance, of which four have been implicated in skin function and/or wound healing. Fibronectin was the most consistently and significantly downregulated (Fig. 2). Like collagen, fibronectin is an ECM glycoprotein predominantly produced by dermal fibroblasts^14^. Also like collagen, fibronectin contains a number of binding sites for growth factors, including FGF, VEGF, and PDGF, which have been shown to promote wound healing and protect against radiation tissue injury^35–38^. As a result, there have been a number of studies demonstrating fibronectin’s role in the promotion of wound healing^15, 18–21^. Though the underlying mechanism has not been fully elucidated, evidence suggests that fibronectin can form a scaffold for epidermal cell migration and modulate cytokines and growth factors in the tissue^15, 19, 21^. Exposure to ionizing radiation has been associated with altered fibronectin levels in other end-organs^22–24^. Additionally, two studies have demonstrated that fibronectin improves cell survival after irradiation *in vitro* ^17, 18^. In our RPPA panel, relative expression of growth factor receptors VEGF-R and PDGF-R did not achieve significance. Of note, our samples were heterogeneous in harvest site, dose of radiation, sex, and time since irradiation. As a result, additional research is required to evaluate how downregulation of fibronectin in irradiated skin affects downstream mediators of cell proliferation and migration.

Other proteins found to be differentially expressed in our RPPA analysis known to be involved in skin function and/or wound healing include collagen VI, p27(Kip1), and androgen receptor. Collagen VI, like fibronectin, is an ECM protein downregulated in irradiated skin (fold-change 0.54). In a study of cell-derived matrices deficient in the protein, collagen VI was critically associated with assembly of the dermal matrix and motility of resident fibroblasts, suggesting that downregulation may limit wound healing^39^. P27(Kip1) is an enzyme inhibitor that controls cell cycle progression and has been shown to play a supervisory role in preventing hyperproliferation during tissue repair^40^. Its downregulation in irradiated skin (fold-change 0.85), then, may contribute to the characteristic hyperkeratosis of radiation-damaged skin. Androgens and the upregulation of androgen receptor expression have been associated with the inhibition of cutaneous wound healing^41, 42^. The effect of its downregulation in irradiated skin (fold-change 0.9), then, is of unclear importance. It is possible that it is a component of the rescue system to compensate for radiation injury, but additional research is needed.

Given the important role of fibronectin in wound healing, the finding that it is expressed at low levels in irradiated wounds prompted us to investigate whether supplementation of the protein would have a significant impact on radiation-induced delayed wound healing. We sought to test this hypothesis using an experimental murine irradiated wound model (Fig. 3A). After confirming that irradiation resulted in fibronectin downregulation and delayed wound healing, we applied topical fibronectin gel or topical vehicle gel to irradiated wounds (Fig. 3B,C). Mice receiving 2.0 mg of topical fibronectin healed significantly faster than control mice (Fig. 3C). Histological analysis demonstrated that this was associated with a decrease in acute inflammatory infiltrate and increase in angiogenesis, resulting in an improved healing score (Fig. 4A–E). These findings are consistent with fibronectin’s known involvement in the storage and release of growth factors and cytokines, which may account for the immunomodulatory and angiogenic effect of topical fibronectin in these wounds. Both TGF-β and SMAD3 have been repeatedly implicated as fundamental mediators of radiation-induced fibrosis. Further, intervention in the TGF-β/SMAD3 pathway results in improved wound healing in irradiated skin^26, 27^. Both molecules were significantly upregulated in irradiated mouse skin, and may serve as a potential target for topical fibronectin’s effects (Fig. 4F,G).

In summary, we are the first group to report that irradiated human skin harvested from patients with severe radiation injury has reduced fibronectin compared to paired non-irradiated skin. Translating these findings to a murine irradiated wound model, topical application of fibronectin to wounds with similarly diminished levels of the protein resulted in significantly improved wound healing. These findings elucidate a potential target for therapy in the prevention and/or treatment of radiation skin injury and irradiated wounds. Given the clinical challenges associated with non-healing radiation wounds, further efforts towards understanding the mechanism of improved wound healing after application of fibronectin will be important in facilitating clinical development of fibronectin-based treatments.

## Materials and Methods

### Reverse Phase Protein Array

In order to evaluate differential expression of proteins in irradiated and non-irradiated skin, we obtained paired samples from patients undergoing post-oncologic reconstruction from March 2012 to March 2014 under a University of Southern California Institutional Review Board-approved protocol. Informed consent was obtained from all participants, and all methods were carried out in accordance with relevant guidelines and regulations. Irradiated tissue was taken from the irradiated field, while non-irradiated tissue was obtained from a distant and non-irradiated site. Subcutaneous tissue was removed from these tissue samples by sharp dissection, after which the samples were flash-frozen in liquid nitrogen and stored at -80 °C prior to protein extraction. Tissues were homogenized to extract protein in radioimmunoprecipitation assay (RIPA) buffer plus phosphatase and protease inhibitors (Sigma-Aldrich, St. Louis, MO) and sonicated to shear genomic DNA. Samples were aliquoted and stored at −80 °C. Samples were diluted to 1 μg/μl in RIPA buffer. RPPA for 210 proteins was performed by the MD Anderson RPPA Core using 30 µg of protein per sample according to lab protocol. Data on irradiated samples were compared to their non-irradiated counterparts using paired t-tests (α < 0.05).

### Western Blot

Paired irradiated and non-irradiated skin samples were obtained from four patients undergoing reconstruction from April 2015 to April 2016. Subcutaneous tissue was removed from the skin by sharp dissection, and 0.5 mg was flash frozen in liquid nitrogen. 200 µl of T-PER Tissue Protein Extraction Reagent (Thermo Fisher Scientific, Waltham, MA) was then added to the tube and the tissue was crushed with a blunt metal instrument and vortexed. The freezing/crushing steps were repeated a minimum of three times until the tissue sample was well liquefied. Each tube was then centrifuged at 14000 rpm for 10 minutes and the supernatant was collected. Protein concentrations were determined using a Bradford assay on a NanoDrop 2000 (Thermo Fisher Scientific, Waltham, MA). 4X Laemmli sample buffer and 2-mercaptoethanol from (Bio-Rad Laboratories, Hercules, CA) were used to dilute each protein sample to a concentration of 1.0 mg/mL. Samples were boiled for 5 minutes and loaded into 4–15% Mini-PROTEAN TGX Precast Protein Gels (Bio-Rad Laboratories, Hercules, CA). Gel electrophoresis was performed at 120 V for 60 minutes with 1X Tris-Glycine Buffer as running buffer (Bio-Rad Laboratories, Hercules, CA). The transfer sandwich was prepared using Immune-Blot PVDF Membrane (Bio-Rad Laboratories, Hercules, CA). The transfer was run at 130 V for 1 hour and 45 minutes. The membrane was washed in 1X TBST with 0.1% Tween 20 solution 3 times for 5 minutes each wash (Bio-Rad Laboratories, Hercules, CA). A 5% milk non-fat dry milk blocking solution was used to block overnight at 4 °C or at room temperature for 2 hours. After blocking, the membrane was washed, cut into two pieces, and blotted separately for fibronectin (catalog #F3648) and beta-actin (catalog #A5441) at concentrations of 1:250 and 1:100, respectively (Sigma-Aldrich, St. Louis, MO). Goat anti-rabbit antibody (catalog #A0545) and goat anti-mouse antibody (catalog #A4416) were used as secondary antibodies at concentrations of 1:500 for the fibronectin and beta-actin blots, respectively (Abcam, Cambridge, UK). The membranes were developed using Clarity ECL Substrate (Bio-Rad Laboratories, Hercules, CA), and film processed in a Konica SRX-101A (Tokyo, Japan).

### RT-PCR

In order to obtain additional confirmation of our RPPA results and translate the findings to an animal model, RT-PCR was performed to evaluate fibronectin expression in both human and murine skin pairs. Human skin samples were the same as those used for Western blotting. Murine skin samples were obtained from mice receiving two 10 Gy radiation doses to the left lateral abdomen, and were harvested 30 days after irradiation. To evaluate the effect of topical fibronectin on TGF-β and SMAD3, known mediators of radiation-induced skin fibrosis, mouse wounds treated with either topical fibronectin or vehicle control were harvested 18 days after treatment. RNA was extracted from these tissues using the TrIzol Plus RNA Purification Kit according to manufacturer protocol (Thermo Fisher Scientific, Waltham, MA) and stored at −80 °C. Primers for mouse and human fibronectin and beta-actin and for mouse TGFβ1 and SMAD3 were purchased from Integrated DNA Technologies (Coralville, IA) and are presented in Table 1. RT-PCR was completed using the Bioline SensiFAST SYBR Hi-ROX One-Step Kit (Taunton, MA) and the Applied Biosystems ABI 7900HT Real-Time PCR System (Foster City, CA). Relative quantification was calculated from the data generated by RT-PCR using the Applied Biosystems SDS 1.5 software.

**Table 1:** Primers utilized for RT-PCR.

Primer	Forward	Reverse
Human Actb	AAGTCAGTGTACAGGTAAGCC	GTCCCCCAACTTGAGATGTATG
Human Fn1	ACCATCTTGTAGGACTGACC	CGTCCTAAAGACTCCATGATCTG
Murine Actb	GTACGACCAGAGGCATACAG	CTGAACCCTAAGGCCAACC
Murine Fn1	GAGCTATCCATTTCACCTTCAGA	TTGTTCGTAGACACTGGAGAC
Murine Tgfb1	CCGAATGTCTGACGTATTGAAGA	GCGGACTACTATGCTAAAGAGG
Murine Smad3	GCGGCACGTAGATAACGTGAG	GAACACCAAGTGCATTACCATC

### Murine Wound Model

Four- to six-week-old male BALB/c mice were purchased from Jackson Laboratories (Bar Harbor, ME). All experimental protocols were approved by the University of Southern California Institutional Animal Care and Use Committee, and all methods were carried out in accordance with relevant guidelines and regulations. The mice were irradiated with two 10 Gy doses with a one-week intervening recuperation period using an X-RAD 320 Irradiator (Precision X-Ray, North Branford, CT) over the left lateral abdomen, just caudal to the forelimb. The mice were then returned to normal housing for 6 weeks prior to wounding and treatment.

A well-described wound model was utilized for this experiment (Fig. 3A)^43^. Briefly, after induction of anesthesia, an 8mm punch biopsy was used to create a full-thickness wound in the left lateral abdomen over the previously-irradiated field. A silicon wound stent was glued to the surrounding skin, then sutured in place with six 5–0 prolene sutures. Treatment or vehicle gel was applied to the wound, and the stent was covered with Tegaderm (3 M, Maplewood, MN). The mice were returned to standard housing. At day 7 the stents were removed with minimal disturbance to the underlying tissue and the wound was allowed to heal without additional intervention. Wound areas were measured using serial photographs at 7-day intervals in ImageJ software (US NIH, Bethesda, MD) by tracing the leading edge of the epithelialized wound. All measurements were standardized against a ruler included within the frame of the photograph. The primary outcome measure was percent wound area relative to the initial wound area, and was calculated by subtracting interval wound area from initial wound area, divided by initial wound area.

### Topical Fibronectin Treatment

A 15% carboxymethylcellulose gel was prepared using medium-viscosity carboxymethylcellulose (VWR, Radnor, PA) and sterile water. Fibronectin gel was created with human plasma fibronectin (Sigma-Aldrich, St. Louis, MO) solubilized in sterile water and gel, formulated to deliver 1.0 mg of fibronectin per 20 mg of gel. Control gel was created with carboxymethylcellulose gel and sterile water alone. Mice received 20 mg of topical fibronectin or control gel on day 0 and day 3.

### Histology

Wound tissue samples were obtained from control and fibronectin-treated mice on day 15 to ensure the presence of a wound gap upon histological examination. The full wound and surrounding skin were harvested along with subcutaneous tissue using sharp dissection, being careful to preserve wound architecture and any granulation tissue within the gap. All samples were fixed in 10% formalin for 24 hours, then placed in 70% ethanol, embedded in paraffin, and sectioned. For hematoxylin and eosin or Sirius Red stains, slides were deparaffinized using serial xylene and ethanol baths then stained according to standard protocol (VWR, Radnor, PA). For immunohistochemistry, sections were deparaffinized using the same method, then processed for antigen retrieval overnight in a sodium citrate buffer (VWR, Radnor, PA) using an Electron Microscopy Sciences Retriever (Hatfield, PA). Slides were blocked for one hour with donkey serum, then washed and stained for myeloperoxidase at 1:250 (R&D Systems, catalog #AF3667, Minneapolis, MN), F4/80 at 1:100 (Abcam, catalog #ab6640, Cambridge, UK) and CD31 at 1:100 (Abcam, catalog #ab28364) overnight at 4 °C. Slides were subsequently washed, then blotted with donkey anti-goat antibody at 1:100 (Abcam, catalog #ab6884), donkey anti-rat antibody at 1:100 (Abcam, catalog #ab102180), and donkey anti-rabbit antibody at 1:100 (Abcam, catalog #ab6801). Slides were washed again, then treated with the VECTASTAIN Elite ABC HRP kit for 30 minutes and IMMPact DAB substrate (Vector Laboratories, Burlingame, CA) until appropriately developed. Slides were counterstained with hematoxylin and mounted. All images were obtained with a Keyence BZ-X700 microscope (Itasca, IL). Using Sulthana *et al*.’s protocol, a wound healing score was calculated for each group^28^.

### Statistics

All statistical analyses were performed with SPSS (IBM, Armonk, NY). Unpaired Student’s *t*-tests were used to compare treatments against control at each measurement interval. Paired Student’s *t*-tests were used to compare relative quantification from RT-PCR.

## Electronic supplementary material

Supplementary Figure

Dataset 1
