# Supplementary material for: Topical Fibronectin Improves Wound Healing of Irradiated Skin
Source: Sci Rep. 2017 Jun 20;7:3876. doi: 10.1038/s41598-017-03614-y (PMC5478660; doi:10.1038/s41598-017-03614-y)
Supplement: Supplementary file 1 — Supplementary Figure [file 41598_2017_3614_MOESM1_ESM.pdf]

Manuscript title: *Topical Fibronectin Improves Wound Healing of Irradiated Skin*

Authors: *Maxwell B. Johnson, Brandon Pang, Daniel J. Gardner, Solmaz Niknam-Benia, Vinaya Soundarajan, Athanasios Bramos, David P. Perrault, Kian Banks, Gene K. Lee, Regina Y. Baker, Gene H. Kim, Sunju Lee, Yang Chai, Mei Chen, Wei Li, Lawrence Kwong, Young-Kwon Hong, Alex K. Wong*

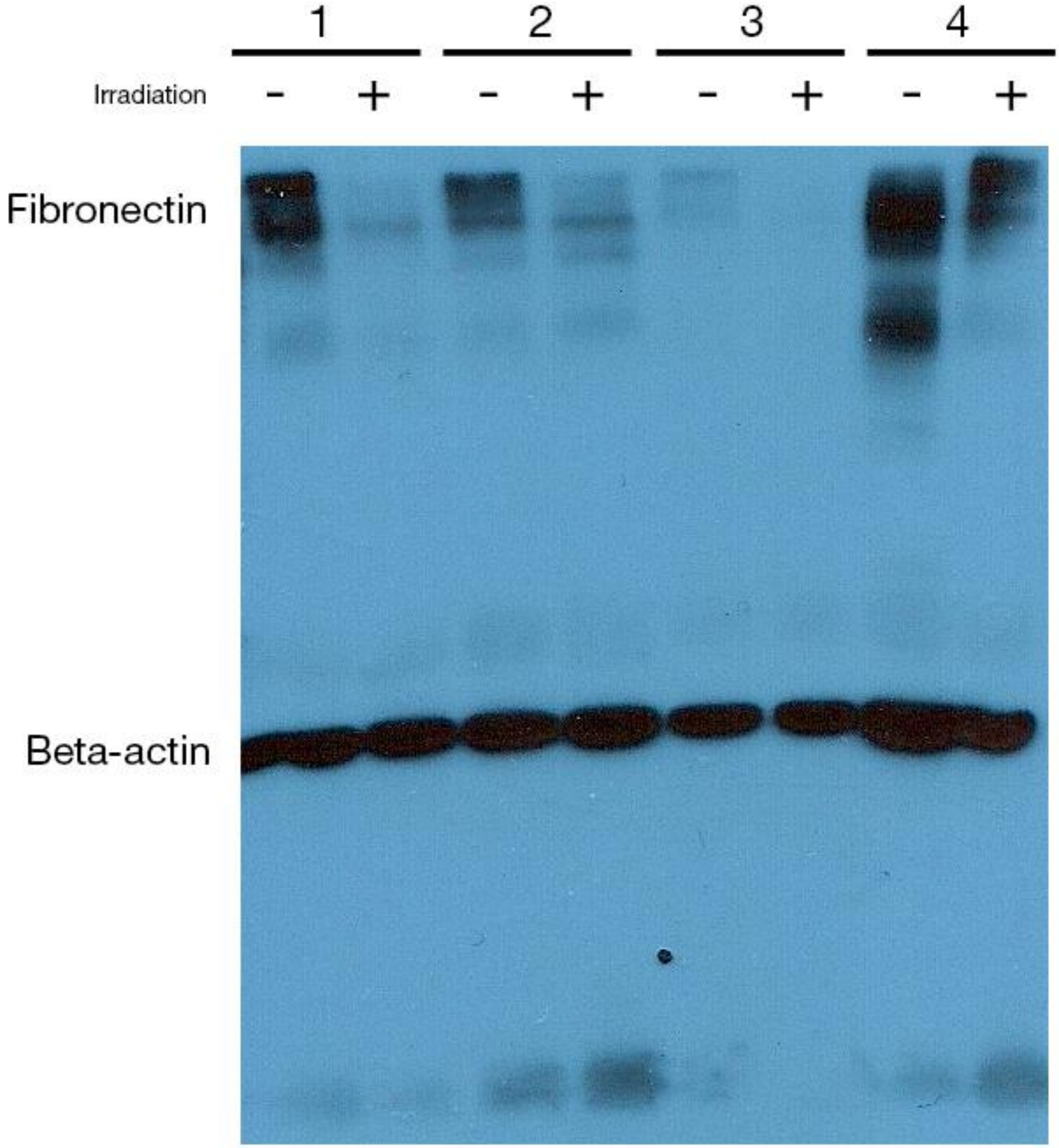

Supplementary Figure 1. Uncropped Western blot of fibronectin expression in four pairs of human skin samples.
